# Longitudinal analysis of exposure to a low concentration of oxytetracycline on the zebrafish gut microbiome

**DOI:** 10.3389/fmicb.2022.985065

**Published:** 2022-09-22

**Authors:** Masood ur Rehman Kayani, Kan Yu, Yushu Qiu, Xiaogang Yu, Lei Chen, Lisu Huang

**Affiliations:** ^1^Department of Infectious Diseases, Xinhua Children's Hospital, Xinhua Hospital, Shanghai Jiao Tong University School of Medicine, Shanghai, China; ^2^National Clinical Research Center for Child Health, The Children's Hospital, Zhejiang University School of Medicine, Hangzhou, China; ^3^School of Life Sciences, Fudan University, Shanghai, China; ^4^Ministry of Education and Shanghai Key Laboratory of Children's Environmental Health, Xinhua Hospital, Shanghai Jiao Tong University School of Medicine, Shanghai, China; ^5^Shanghai Institute of Immunology, Shanghai Jiao Tong University School of Medicine, Shanghai, China

**Keywords:** gut microbiome, oxytetracycline, longitudinal analysis, goblet cells, low concentration, antibiotic exposure, environmental pollution

## Abstract

Oxytetracycline, a widely produced and administered antibiotic, is uncontrollably released in low concentrations in various types of environments. However, the impact of exposure to such low concentrations of antibiotics on the host remains poorly understood. In this study, we exposed zebrafish to a low concentration (5,000 ng/L) of oxytetracycline for 1 month, collected samples longitudinally (Baseline, and Days 3, 6, 9, 12, 24, and 30), and elucidated the impact of exposure on microbial composition, antibiotic resistance genes, mobile genetic elements, and phospholipid metabolism pathway through comparison of the sequenced data with respective sequence databases. We identified *Pseudomonas aeruginosa*, a well-known pathogen, to be significantly positively associated with the duration of oxytetracycline exposure (Adjusted *P* = 5.829e^−03^). Several tetracycline resistance genes (e.g., *tetE*) not only showed significantly higher abundance in the exposed samples but were also positively associated with the duration of exposure (Adjusted *P* = 1.114e^−02^). Furthermore, in the exposed group, the relative abundance of genes involved in phospholipid metabolism had also decreased. Lastly, we characterized the impact of exposure on zebrafish intestinal structure and found that the goblet cell counts were decreased (~82%) after exposure. Overall, our results show that a low concentration of oxytetracycline can increase the abundance of pathogenic bacteria and lower the abundance of key metabolic pathways in the zebrafish gut microbiome that can render them prone to bacterial infections and health-associated complications.

## Introduction

Oxytetracycline (OTC) is a routinely used antibiotic for animal disease control as well as for agricultural feed additives globally due to its affordability, availability, and efficiency (Li et al., 2019). OTC is typically poorly absorbed, and according to an estimate, nearly 30–90% of the antibiotic could be excreted in the environment (Wang et al., 2018). As a consequence of its widespread application, OTC has been detected from diverse environmental settings (Xu et al., 2021). For instance, OTC has been detected at concentrations of ~7,993 ng/L in a Brazilian farm (Monteiro et al., 2016), 3,138 ng/L in the United States (Zhang et al., 2013), and >5,000 ng/L in China (Bu et al., 2013). Although these concentrations are much lower than clinical doses, their frequent presence in the environment can lead to the emergence of antibiotic resistance in bacteria or promote the dissemination of antibiotic resistance genes in exposed flora (Arabpour and Nezamzadeh-Ejhieh, 2016; Xu et al., 2016; Belkacem et al., 2017). Therefore, these low concentrations (LCs) of antibiotics can cause serious problems for food safety and human health but their impact remains poorly characterized. In recent years, certain efforts have explored the hazardous effects of exposure to LCs of OTC. For instance, it has been demonstrated that OTC exposure in aquatic environments can cause damage to thyroid function (Yu et al., 2020) and oxidative stress toxicity (Zhou et al., 2018). However, it is highly critical to developing a comprehensive understanding of the impact of LCs on the exposed life forms through further studies.

The gut microbiome is highly important for the hosts for the modulation of metabolic, immune, and neuroendocrine pathways. Dysbiosis in the gut microbiome, driven by antibiotics, may cause malfunctions in the host and ultimately result in the progression of disease (Kho and Lal, 2018). It can be hypothesized that the LCs of OTC (or any other antibiotic released in the environment) could also result in similar dysbiosis of the intestinal microbiome and produce adverse effects. 16S rRNA gene sequencing-based microbial community analyses showed no significant change in the zebrafish gut microbial diversity after exposure to 100–420 ng/L OTC (Almeida et al., 2019; Li et al., 2020; Kayani et al., 2021). Using shotgun metagenomics, our previous study showed that the enrichment of multiple flavobacterial species occurred after exposure to OTC (1,000–5,000 ng/L) in the zebrafish (Kayani et al., 2021). Flavobacterial species are responsible for devastating loss in wild and farmed fish stocks around the world, as they are associated with several diseases in fish (Loch and Faisal, 2015). In addition, it is also plausible that OTC exposure affects the antibiotic resistance genes (ARGs) and increases their chances of horizontal gene transfer (HGT) *via* mobile genetic elements (MGEs) during exposure. However, the concomitant understanding of the impact associated with duration of exposure to low concentration of OTC on the composition of ARGs as well as their interplay with MGEs remains unknown. Therefore, a longitudinal analysis must be performed for developing a holistic understanding of the impact of exposure to LCs on microbial taxonomic structure, metabolic functions as well as ARG-MGE interactions.

The intestinal epithelium is crucial for preserving gut homeostasis. It acts both as a physical barrier and as a coordinating hub for the immune response and crosstalk between microbes and the immune cells (Allaire et al., 2018). One of the most important contributors, the intestinal goblet cells, secrete the mucus that separates the commensal bacteria from the host epithelium (Pelaseyed et al., 2014). Antibiotic exposure has been shown to reduce the goblet cell count that in turn results in tumor development (Kaur et al., 2018). In a previous study, a high concentration of OTC resulted in increased intestinal inflammation and damaged intestinal goblet cells (Zhou et al., 2018), but the impact of exposure to a low concentration of OTC on the mucus morphology, especially on the goblet cell counts, remains unknown.

In this study, we performed a longitudinal collection of samples, followed by shotgun metagenomic sequencing and analysis, to characterize the impact of exposure to low concentration of OTC on the zebrafish gut microbiome. Our primary objectives in this study were: (i) characterization of the impact of OTC exposure on the diversity and composition of zebrafish gut microbiome and determining the associations between microbes and exposure duration, (ii) elucidating how the ARGs and MGEs associate with each other after OTC exposure, (iii) characterizing the impact of OTC exposure on the microbial phospholipid biosynthesis pathway, and (iv) determining the impact of OTC exposure on the zebrafish intestinal physiology. We hypothesized that an increase in the duration of OTC exposure would result in the gradual accumulation of pathogenic microbes in the gut microbiome, increase the strength of associations between the ARGs and MGEs, and more severely hamper the ability of microbes to synthesize phospholipids in the exposure group. To this end, longitudinal samples were collected from the exposed and control samples, followed by shotgun metagenomic sequencing and bioinformatics analysis, to determine the causal effects of OTC exposure.

## Materials and methods

### Zebrafish husbandry and antibiotic exposure

Adult wild-type zebrafish (strain AB), a widely used model organism in ecotoxicology research as well as for determining the harmful impacts of exposure to environmental toxins in the health and disease (Bambino and Chu, 2017), was purchased from the Ministry of Education and Shanghai Key Laboratory of Children's Environmental Health, Xinhua Hospital. Furthermore, the gut microbiome in adults is more stable, than in juveniles in which the microbiome is in process of assembly and succession. Therefore, we used adult zebrafish to avoid bias caused by variance in the microbiome composition due to zebrafish development. In this study, zebrafish at 120 days post-fertilization (dpf) were exposed to 5,000 ng/L of OTC. The same number of zebrafish (*n* = 20) was kept in untreated water as a control group. All treatments were performed in triplicate. The maximum tank volume was 20 L whereas a 12:12 h light: dark light cycle, and twice daily feeding (with brine shrimp) were maintained during the study period. Half of the solution in the water tank was replaced daily, and the corresponding concentration of antibiotic was added immediately. The water temperature and pH value in the water tank were kept at 28 ± 1°C and 6.8–7.5, respectively. OTC (CAS: 615 3-64-6) was purchased from Yuanmu Biotechnology Co., Ltd. (Shanghai, China). The stock solution was prepared at 50 mg/L as described elsewhere (Walter et al., 2019).

### Collection of fecal material, DNA extraction, and sequencing

**Figure 1:**
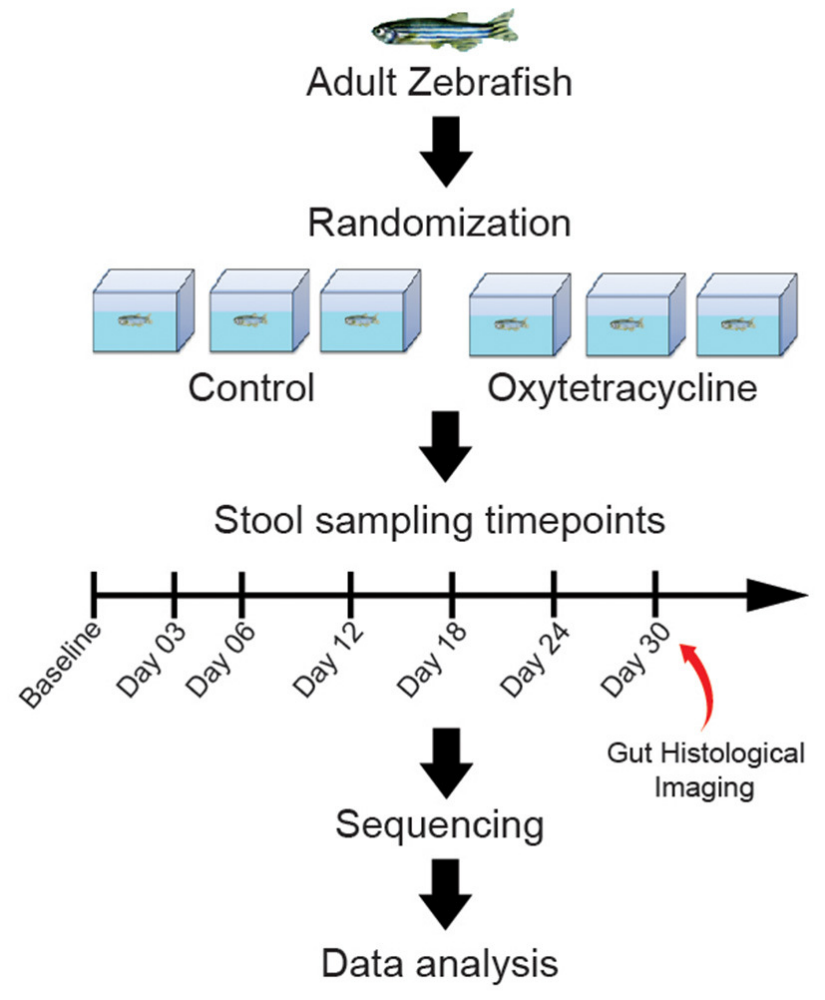
Graphical abstract of the study design. Zebrafish were grouped into control and exposure categories, and longitudinal samples were collected at Baseline, D03, D06, D12, D18, D24 and D30. The exposure group was exposed to 5000 ng/L of OTC for 30 days whereas no exposue was performed in the control group. The longitudinally collected samples were sequenced and data analysis was performed.

Fecal samples were collected before exposure (Baseline) and longitudinally on days 3, 6, 12, 18, 24, and 30 after exposure to OTC (Supplementary Table 1). Similarly, fecal samples were also collected at identical time points from the control group (Figure 1). We believe that sampling at these time points can allow us to elucidate the temporal patterns of changes in the gut microbiome diversity and composition caused by OTC exposure. Sample collection was performed as described elsewhere (Gaulke et al., 2019) while metagenomic DNA was extracted following a previously established protocol (Gilchrist et al., 2007) using Qiagen PowerFecal Kit (Catalog No. 12830–50; Qiagen, Hilden, Germany), following the manufacturer's protocol. The extracted DNA was stored at −80°C until further usage.

Five hundred ng of DNA was used to construct the paired-end metagenomic libraries with the Nextera DNA Flex Library Prep kit (Illumina, CA, US), following the manufacturer's protocol. Briefly, fragmentation and adapter ligation of the DNA was performed, followed by polymerization of the adapter-ligated library and purification of the amplified library. Illumina Hiseq X Ten (Illumina, CA, US) sequencer was used for metagenomic sequencing of the purified library with paired-end (PE) reads of ~150 bp per read-end.

### Read preprocessing, *de novo* assembly, and gene catalog construction

The sequence read quality was visualized using FastQC (v0.11.8) whereas the reads were preprocessed for the removal of low-quality sequences (quality lower than Q20), adapter sequences, and ambiguous bases (N) using TrimGalore (v0.5.0) (Andrews, 2010; Krueger, 2015). Furthermore, reads were mapped to the zebrafish reference genome (GCA_000002035.4) and the Human genome (hg38) using BMTagger (v1.1.0; Rotmistrovsky and Agarwala, 2011), and successfully mapped reads were removed from downstream analyses.

Metagenomic *de novo* assembly was performed using MEGAHIT assembler (v1.1.4; Mayer et al., 2015) with *k*-mers ranging from 29 to 149 (–k-min 29, –k-max 149), a *k*-mer step size of 10 (–k-step 10) and minimum contig length of 200 bp (–min-contig-len 200). Prodigal (v2.6.3) (Hyatt et al., 2010) was used for the prediction of genes using the metagenomic procedure (-m) and translation Table 11. Genes smaller than 100 bp were excluded using Seqtk (v1.3) (Li, 2012) whereas CD-HIT-EST (v4.8.1) was used for the removal of redundancy (Li and Godzik, 2006) with sequence identity threshold of 95% (-c 0.95), alignment coverage of 90% for the shorter sequence (-aS 0.9) and word length of 8 (-n 8). These non-redundant genes were used for functional analyses.

### Taxonomic and functional analyses

High-quality PE reads were used for performing the taxonomic analysis using Kaiju (v1.6.3) (Menzel et al., 2016). Briefly, each PE read was assigned to a taxon in the NCBI taxonomy by comparing it to the representative set of genomes from the proGenomes database and viruses from the NCBI RefSeq database (Mende et al., 2016).

For functional analyses, reference ARGs were downloaded from the comprehensive antibiotic resistance gene database (CARD) (Alcock et al., 2020) whereas non-redundant MGEs (Pärnänen et al., 2018) were obtained from https://github.com/KatariinaParnanen/MobileGeneticElementDatabase and used to identify mobile genetic elements. In addition, a custom database of genes involved in phospholipid metabolism (biosynthesis and degradation) was created after genes associated with this pathway were identified through a literature search and subsequently downloaded from the NCBI Identical Protein Group database (Supplementary Table 7). Quantification of ARGs, MGEs and genes involved in phospholipid biosynthesis was performed using ShortBRED (v0.9.3) (Kaminski et al., 2015). Briefly, for each of the ARG, MGE and phospholipid metabolism gene databases, protein markers were produced from the non-redundant gene catalog using the ShortBRED_Identify script. These marker sets were then quantified with the high-quality reads of each sample using the ShortBRED_Quantify script to obtain the relative abundance of markers of ARGs, MGEs and genes involved in phospholipid biosynthesis.

### Histopathological examination

For performing the histopathological examination, 3 fish were collected from the exposed and control groups at Day 30. Zebrafish were sacrificed and the 0.5 cm long foregut was fixed in 4% paraformaldehyde for 24 h. Subsequently, the sample was transferred to ethanol for dehydration, then transferred to xylene, and embedded in paraffin. The foregut was cut transversely into 5 mm sections and mounted on glass slides. Hematoxylin-eosin (H & E) were used to stain the slides, which were visualized under a microscope (Nikon, Eclipse, TS100). Image-pro plus 6.0 (Media Cybernetics, Inc., Rockville, MD, USA) was used for counting the number of goblet cells in the intestinal tissue.

### Comparative and statistical analyses

Alpha and beta diversities were calculated with the vegan package of R language (Oksanen et al., 2007) whereas the differences in alpha diversity were tested with the Wilcoxon test. The identification of differentially abundant taxa was performed using the Linear discriminant analysis Effect Size (LEfSe) (Segata et al., 2011). The threshold on the logarithmic linear discriminant analysis (LDA) score for discriminative features was set to >4.0. The hierarchical All-against-All association (HAllA) tool was used for identifying the Spearman's correlation (between ARGs and MGEs) and determining its strength (Rahnavard et al., 2017). MaAsLin2 was used to identify associations between metadata and abundance profiles (Species, ARGs, and MGEs) (Mallick et al., 2021). For all statistical tests, *P* < 0.05 was considered statistically significant. Benjamini and Hochberg's false discovery rate (FDR) controlling procedure was used for correcting *P*-values (in certain cases) and a corrected *P*-value of <0.1 was considered statistically significant.

## Results

### Diversity and structure of the zebrafish gut microbiome

**Figure 2:**
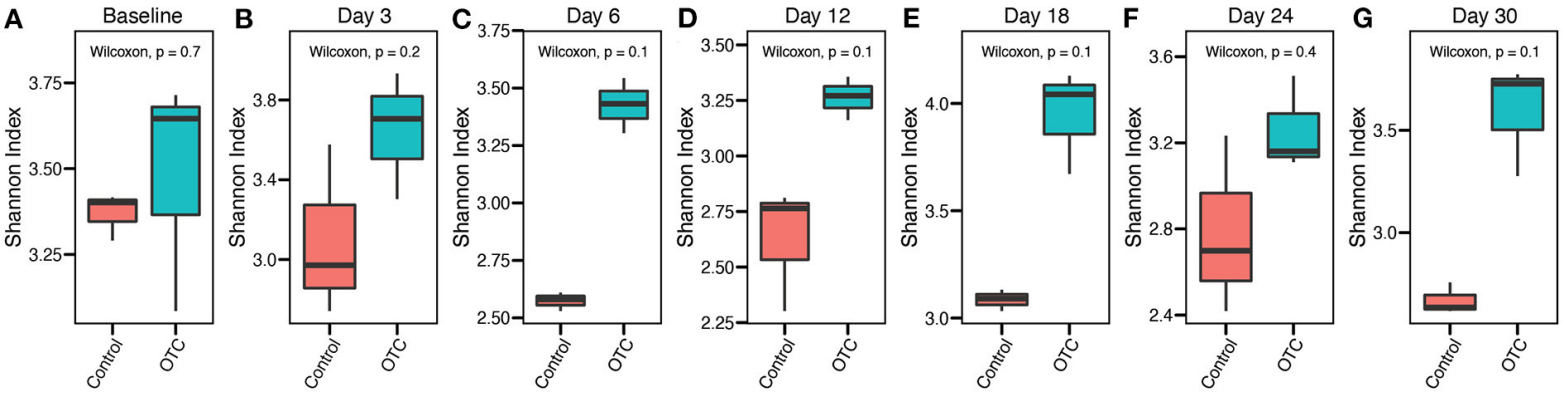
Trends in the α-diversity of the zebrafish gut microbiome during OTC exposure. **(A–G)** α-diversity (Shannon Index) comparisons between the control and OTC exposure groups at different timepoints during the one-month exposure window are shown. These results indicated the differences in the α-diversity were not statistically significant (Wilcoxon test, *P* > 0.05) between the two groups at all of the sampled timepoints. However, the mean α-diversity remained relatively higher in the OTC exposure group.

We first assessed the impact of exposure to OTC on the microbial diversity of the zebrafish gut microbiome. A comparison of alpha diversity at baseline indicated that the differences between the control and exposed groups were not statistically significant (Wilcoxon's test, *P* > 0.05). Exposure to OTC (Day 3 and onwards) also did not result in significant shifts in the microbial alpha diversity (Wilcoxon's test, *P* > 0.05) compared with the control group at respective time points. We confirmed this trend using multiple different alpha diversity metrics, including Shannon, Simpson, and Inverse Simpson (Figure 2 and Supplementary Figure 1).

**Figure 3:**
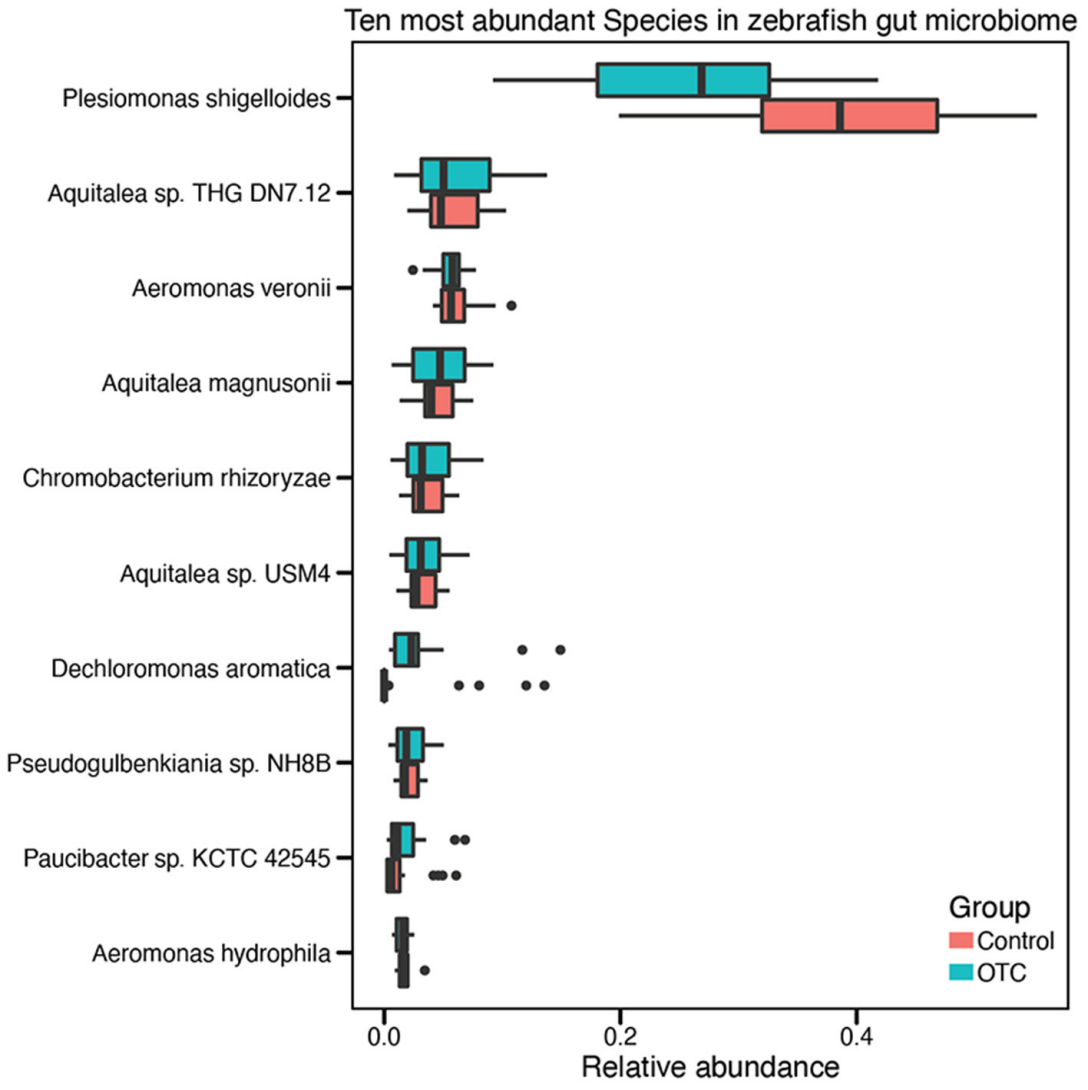
Distribution of the ten highly abundant microbial species in the zebrafish gut microbiome. Taxonomic analysis of the sequencing reads identified more than 300 microbial species in the zebrafish gut microbiome. However, for visual convenience, here we shown only the top ten microbial species. The complete list of species and their relative abundances are provided in Supplementary Table 2.

We determined the community composition of the zebrafish gut microbiome using Kaiju and proGenomes and NCBI RefSeq databases (details described in “Materials and Methods”). We identified 22 different phyla in the zebrafish gut microbiome. Among these phyla, Proteobacteria were overwhelmingly abundant with a mean relative abundance of 83 ± 6% among all samples, followed by Bacteroidetes (7%), Fusobacteria (2.6%), Firmicutes (2.5%), and Actinobacteria (2.2%). The identified microbial taxa were contained in 40 different classes. Among these, Gammaproteobacteria (40.1 ± 0.9%), Betaproteobacteria (36.2 ± 1.2%), Alphaproteobacteria (40.1 ± 0.9%), Flavobacteriia (0.7%) and Fusobacteriia (0.3%) were the five most abundant classes. Distribution of taxa at species level indicated the presence of >300 microbial species. The minimum and the maximum number of identified species in a single sample were 64 and 149, respectively. *Plesiomonas shigelloides* (32 ± 1.1%), *Aquitalea* sp. THG-DN7.12 (5.9 ± 3.2%), *Aeromonas veronii* (5.9 ± 1.5%), *Aquitalea magnusonii* (4.43 ± 2.1%), *Chromobacterium rhizoryzae* (3.67 ± 1.9%), *Aquitalea* sp. USM4 (3.23 ± 1.6%), *Dechloromonas aromatica* (2.47 ± 3%), *Pseudogulbenkiania* sp. NH8B (2.18 ± 1%), *Paucibacter* sp. KCTC 42545 (1.59 ± 1%), and *Aeromonas hydrophila* (1.59 ± 0.5%) were identified as the top 10 most abundant microbial species. In contrast, *Planctomyces* sp. SH-PL62, *Methylibium petroleiphilum, Janthinobacterium* sp. Marseille, *Variovorax boronicumulans*, and *Pedobacter* sp. PACM 27299 were among the least abundant microbial species in the zebrafish gut microbiome (Supplementary Table 2 and Figure 3).

Next, we assessed the compositional differences between the control and OTC exposure groups. The Bray-Curtis dissimilarity and subsequent clustering of the samples on the basis of calculated distances indicated that most of the control and OTC exposed samples clustered separately (Supplementary Figure 2). We further performed community-level multivariate comparisons using PERMANOVA which highlighted significant compositional differences between the two groups (*R*^2^ 0.20217, *P* < 0.001) and different time points (*R*^2^ 0.37225, *P* < 0.001). These results (Table 1) indicate that OTC was unable to significantly impact the alpha diversity but it resulted in temporal shifts in the community composition of the zebrafish gut microbiome.

**Table 1:** PERMANOVA results based on Bray-Curtis distances using the abundance data for zebrafish gut microbiome.

	**Df**	**Sum Sq**	**Mean Sq**	**F Model**	***R* ^2^**	***P-*value**
Timepoint	6	1.11718745	0.18619791	8.00952643	0.37225342	0.001
Group	1	0.60674699	0.60674699	26.0999496	0.20217166	0.001
Timepoint:Group	6	0.62629555	0.10438259	4.49014242	0.20868536	0.001
Residuals	28	0.65091756	0.02324706	NA	0.21688956	NA
Total	41	3.00114755	NA	NA	1	NA

Df, Degrees of freedom; Sum Sq, sum of squares; Mean Sq, Mean of squares.

### Correlation between microbial species and exposure duration

**Figure 4:**
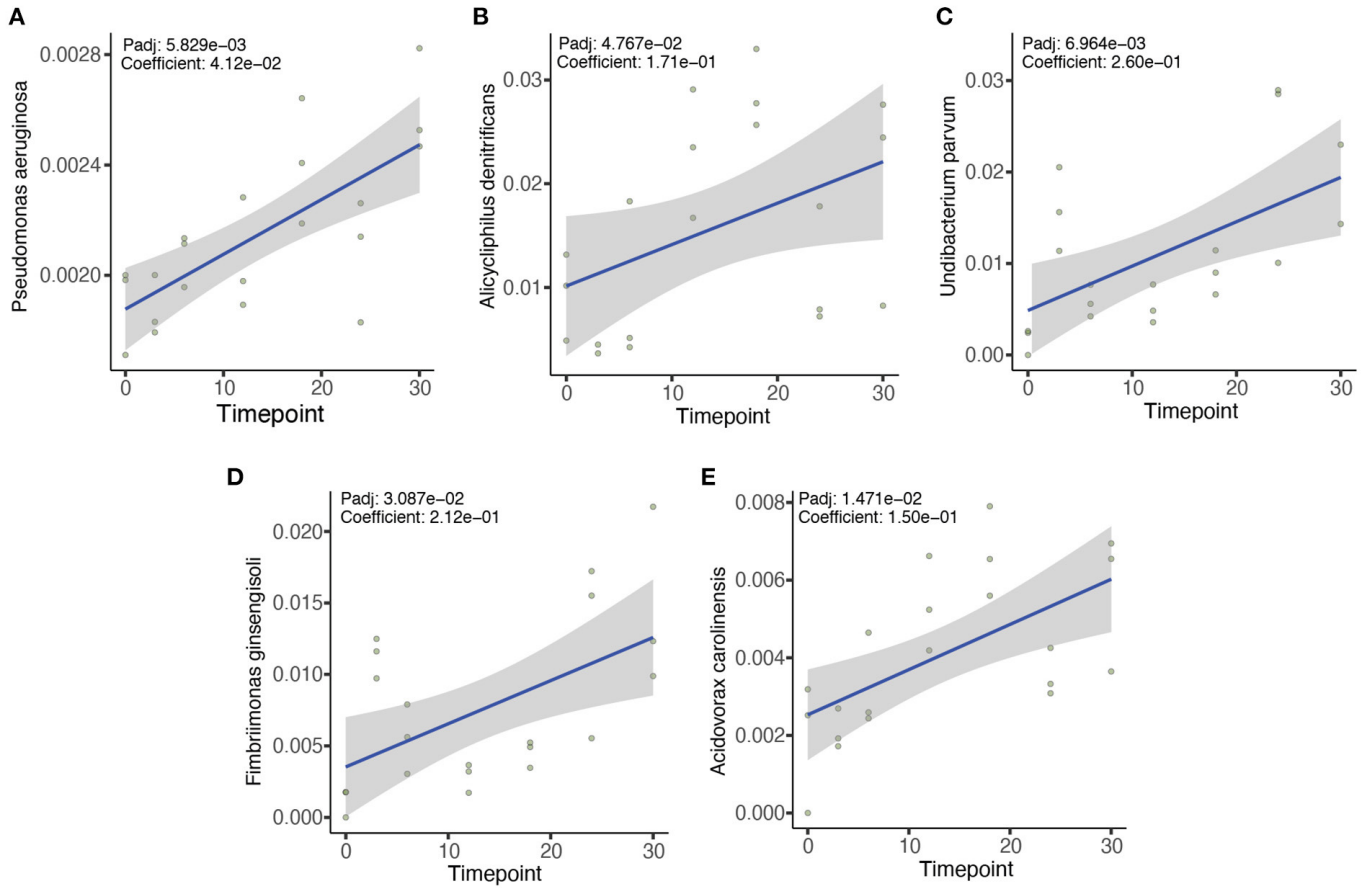
Five most significant associations between microbial species and the duration of OTC exposure. MaAsLin2 was used to evaluate the association between different microbial taxa with the duration of OTC exposure. We identified numerous positive and negative associations between the microbes and OTC exposure (Supplementary Table 4). However, *Pseudomonas aeruginosa* **(A)**, *Alicycliphilus denitrificans* **(B)**, *Undibacterium parvum* **(C)**, *Fimbriimonas ginsengisoli* **(D)**, and *Acidovorax carolinensis* **(E)** showed the strongest positive associations. Benjamini and Hochberg false discovery rate (FDR) controlling procedure was performed for the correction of *P-*values (*P*adj) and only *P*adj < 0.1 were considered statistically significant.

We then sought to determine the association of the microbial species with the duration of the exposure using MaAsLin2 (Mallick et al., 2021). Our results indicated that 132 different microbial species from the zebrafish gut microbiome showed positive or negative associations with the duration of the OTC exposure. Among the positively correlated species (FDR corrected *P-*value (*P*adj < 0.1), *Pseudomonas aeruginosa, Alicycliphilus denitrificans, Undibacterium parvum, Fimbriimonas ginsengisoli*, and *Acidovorax carolinesis* were identified in the majority (>95% samples) of the exposed samples to be significantly positively associated with the duration of exposure (Figures 4A–E). In addition, *Rhodoferax saidenbachensis, Chryeolinea* sp. KIS68-18, *Massilia* sp. NR4-1, *Massilia putida, Acidovorax* sp. T1, and *Janthinobacterium agaricidamnosum* were all also significantly positively associated with the duration of OTC exposure (Supplementary Table 3). In contrast, *Aquaspirillum* sp. LM1, *Enterobacter asburiae, Janthinobacterium* sp. B98, *Aeromonas hydrophila, Enterobacter cloacae, Enterobacter roggenkampii* and *Flavobacterium* sp. HYN0086 exhibited significant negative association with the exposure duration (Supplementary Table 3).

In contrast with the OTC exposure group, we did not identify any significant positive or negative associations for the above-mentioned species in controls. The associations identified in the control group were mostly negative and insignificant. These included several lesser known microbial species such as *Rhodobacteraceae* bacterium QY30, *Tabrizicola* sp. K13M18, *Gemmobacter* sp. HYN0069 and *Shinella* sp. HZN7 (Supplementary Table 4).

### Identification of differentially abundant species

Next, we sought to identify the differentially abundant microbial species between the control and OTC groups as well as determine the possible temporal patterns in their differential abundance. For this purpose, we used LEfSe with α-value of 0.05 and an LDA score of >4 as the threshold. At the baseline, control and OTC did not show any differentially enriched/depleted species whereas at D03 only *Paucibacter* sp. KCTC 42545 showed depletion after OTC exposure. *Plesiomonas shigelloides* was significantly enriched in the controls at D06, D12 and D18. Furthermore, *Flavobacterium anhuiense* was also highly enriched at D12, D18 and D24 and *Chromobacterium rhizoryzae* was significantly enriched at D24 and D30 in the control group (Table 2).

**Table 2:** Differentially abundant (enriched) microbial species identified using LEfSe (LDA score ≥ 4.0 as threshold).

**Species**	**Group**	**Timepoint**
*Paucibacter* sp. KCTC 42545	Control	D03
*Fusobacterium mortiferum*	Control	D06
*Plesiomonas shigelloides*	Control	D06, D12, D18
*Aquitalea* sp. THG-DN7.12	OTC	D06, D12
	Control	D24, D30
*Aquitalea magnusonii*	OTC	D06, D12
	Control	D24, D30
*Chromobacterium rhizoryzae*	OTC	D12
	Control	D24, D30
*Aquitalea* sp. USM4	OTC	D12
	Control	D24, D30
*Alicycliphilus denitrificans*	OTC	D12, D18
*Pseudogulbenkiania* sp. NH8B	OTC	D12
*Flavobacterium anhuiense*	Control	D12, D18, D30
*Dechloromonas aromatica*	Control	D18, D24, D30
*Shewanella* sp. FDAARGOS 354	Control	D18
*Undibacterium parvum*	OTC	D24, D30
*Spirosoma aerolatum*	Control	D30

In addition to the above-mentioned species, several members of the gut microbiome were enriched in OTC exposure group. *Alicycliphilus denitrificans* was more abundant in the OTC group in contrast with control at D12 and D18 while *Dechloromonas aromatica* exhibited significantly higher abundance at D18, D24 and D30. *Undibacterium parvum* is another microbial species that was enriched in OTC exposure groups (D24 and D30). Interestingly, for certain members of the zebrafish gut microbiome that demonstrated enriched abundance at initial timepoints (D06, D12) of exposure, the relative abundances were restored at D24 and D30. These microbes included *Aquitalea magnusonii, Aquitalea* sp. THG-DN7.12, and *Aquitalea* sp. USM4 (Table 2).

### Impact of OTC exposure on ARGs and MGEs

**Figure 5:**
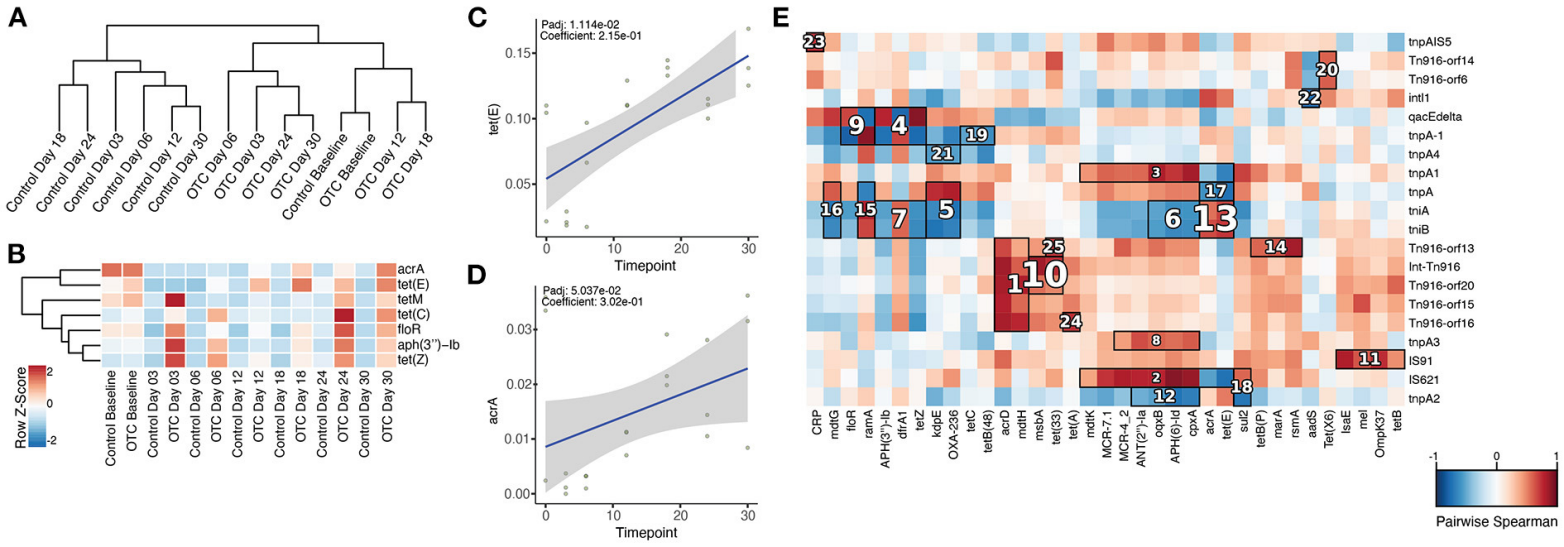
Composition of antibiotic resistance genes and their association with mobile genetic elements in the zebrafish gut microbiome. **(A)** Dendrogram based on the composition of ARGs indicates that the samples from control and OTC exposure groups clustered separately. **(B)** Relative abundance of the ARGs identified to be highly abundant in the OTC exposure group. **(C,D)** *tet(E)* and *acrA* showed significant positive correlation with the duration of exposure to OTC. **(E)** The hallagram shows clusters of significantly correlated antibiotic resistance genes and mobile genetic elements. Correlations were computed using Spearman's coefficient, Benjamini and Hochberg false discovery rate (FDR) controlling procedure was performed for the correction of *P* values (*P*adj) and only *P*adj < 0.1 was considered statistically significant. Clusters are ranked according to their significance (*P*adj) i.e., 1 represents the most significant correlation and vice versa. Here, only top 25 correlation clusters are shown while non-significant correlations and clusters are masked.

We then explored the impact of OTC exposure on the composition and abundance of ARGs as well as on MGEs. To achieve this, we first generated a set of *de novo* assembled contigs and constructed a catalog of non-redundant genes for the zebrafish gut microbiome as described in the Methods. By performing a search against CARD, we identified 49 different ARGs in the zebrafish gut microbiome. Based on the relative distribution of these ARGs, controls and OTC exposed samples demonstrated obvious differences. The baseline of the two sample groups formed one cluster whereas the remaining control and exposed samples clustered separately (Figure 5A). Composition-wise, the zebrafish gut resistome constituted several important ARGs: including *acrAD, cpxA, emrBR, floR, mdtBGHKM, sul2*, and multiple different tetracycline resistance genes (e.g., *tet33, tetB, tetC*, and *tetE*). Distribution according to the relative abundances indicated that *ramA* (3.2% mean relative abundance), *aadS* (2.45%), *floR* (1.39%), *tetE* (0.96%), and *tetZ* (0.69%) were the top 5 most abundant ARGs in the zebrafish gut resistome (Supplementary Table 5).

For determining the impact of OTC exposure on the zebrafish gut resistome and its association with the duration of exposure, we applied the linear models using MaAsLin2. The results indicated that the relative abundances of several ARGs were significantly associated with the duration of exposure. *tetZ, oqxA, floR, aph(3”)-Ib, tetC, tetE, tetM*, and *acrA* were all significantly enriched (*P*adj < 0.1) in OTC exposure group in contrast with the control (Figure 5B). However, only *tetE* (*P*adj = 0.01, Correlation coefficient = 0.21) and *acrA* (*P*adj = 0.05, Correlation coefficient = 0.30) showed significant positive correlation with the duration of exposure (Figures 5C,D). In contrast, these associations were not observed in the control group.

We further characterized the distribution of MGEs in the zebrafish gut microbiome. This was achieved by comparing the non-redundant gene catalog with a mobilome database (as described in the methods) which resulted in the identification of 32 different MGEs. Among these, *tnpA, qacEdelta, intl1, IS91*, and *tniA* were the five most abundant MGEs (Supplementary Table 6). Furthermore, hierarchical clustering separated the controls from the exposed samples (Supplementary Figure 3A). Interestingly, the *tnpA2* gene showed a significant positive correlation with the duration of exposure as its relative abundance increased over the exposure time when compared with the baseline. When contrasted, we observed no significant positive or negative correlations with time in the control group (Supplementary Figure 3B).

Next, we characterized the possible co-occurrence patterns of ARGs and MGEs by performing clustering based on Spearman's correlation using HAIIA (as described in the Methods). We identified 52 different clusters of significant correlation (*P*adj < 0.1) between ARGs and MGEs that were further filtered to obtain the 25 strongest associations. Among these clusters, multiple tetracycline resistance genes (*tet33, tetA, tetB, tetE, tetP, tetZ*, and *tetX6*) formed clusters of positive co-occurrence with various MGEs that included *IS91, tniA, tniB*, and *qacEdelta* (Figure 5E).

### Phospholipid biosynthesis and goblet cell counts significantly altered by OTC exposure

**Figure 6:**
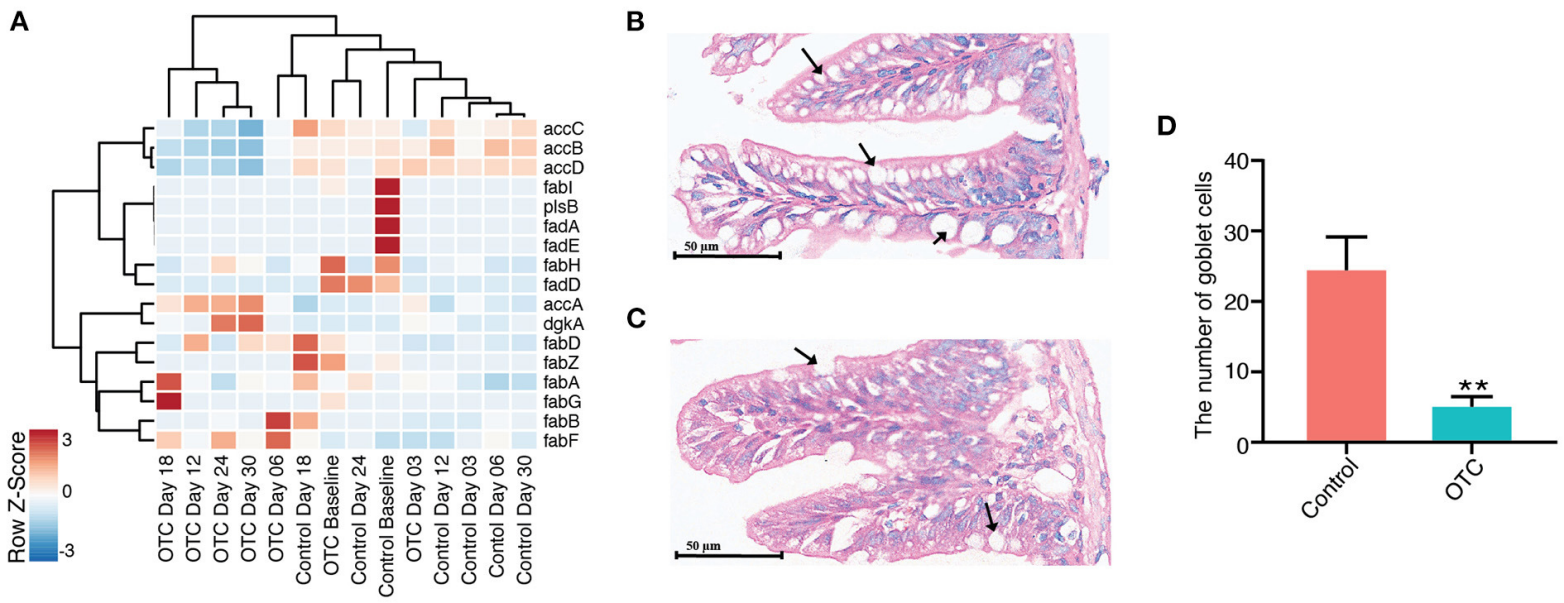
Impact of OTC exposure on microbial phospholipid metabolism and zebrafish intestinal goblet cells. **(A)** Heatmap showing the relative abundance of major genes involved in phospholipid synthesis or degradation. Majority of the genes involved in phospholipid synthesis (e.g., *accB, accC, accD*, and *fabI*) were highly abundant in control whereas we observed decreased abundance of these genes after OTC exposure. Furthermore, no major differences were observed in the relative abundance of phospholipid degrading genes (e.g., *fadA, fadB, fadD, fadE, and fadL*) in control and OTC exposure groups. **(B–D)** Histological examinations in zebrafish gut following exposure to OTC for 30 d (200× magnification, scale bars 50 μm). **(B)** Control group, **(C)** OTC exposure group and **(D)** The number of goblet cells (as indicated by the black arrows) in zebrafish gut (*n* = 5). The data presented are the mean ± SEM of triplicates. Significant differences between OTC groups and the corresponding control group are indicated by *P < 0.05, **P < 0.01.

We hypothesized that OTC exposure could also impact phospholipid biosynthesis in the zebrafish gut microbiome. For evaluating this, we used the non-redundant gene catalog for performing a similarity search against a customized database of genes involved in the metabolism of phospholipids (see Methods for details). Our results indicated that OTC exposure significantly altered the ability of phospholipid biosynthesis in the zebrafish gut microbiome. This is supported by the gradual decrease in the relative abundance of the phospholipid synthesizing genes observed during OTC exposure. Phospholipid biosynthesis genes including *accB, accC, accD*, and *fabI* showed higher abundance in the baseline samples. However, in the post-exposure samples (D03 and beyond) their abundance significantly (*P*adj < 0.1) decreased (Figure 6A). *accB* was the most negatively affected gene after the exposure (Correlation coefficient = −0.27, *P*adj = 0.000029), followed by *accD* (Correlation coefficient = −0.21, *P*adj = 0.00003) and *accC* (Correlation coefficient = −0.20, *P*adj = 0.00008). Surprisingly, *accA* showed significant positive correlation in contrast with the baseline (Correlation coefficient = 0.063, *P*adj = 0.00008). In the control group, we did not observe significant alterations in relative abundances of the genes (in samples collected at D03 and onwards) involved in phospholipid biosynthesis when contrasted with baseline (data not shown). In addition to the genes involved in the synthesis of phospholipids, our custom database also included several genes involved in the degradation of the phospholipids (*fadA, fadB, fadD, fadE, and fadL*). However, we did not observe an enrichment of these phospholipid catabolizing genes after OTC exposure or in the control group. In order to detect the influence of exposure to OTC on the intestinal structure, H&E staining was used to determine the intestinal structure. After exposure, goblet cells in the intestine became unclear in morphology and their number was significantly reduced i.e., by 81.4% (Figures 6B–D).

## Discussion

Low concentrations of antibiotics are often identified in various environments. However, their impact on the exposed life remains poorly understood. Our work focuses on determining the temporal dynamics of exposure to low concentration of oxytetracycline on zebrafish gut microbiome. Through longitudinal sampling, we show that the low concentration of oxytetracycline results in the enrichment of pathogenic bacteria and multiple antibiotic resistance genes in the zebrafish gut microbiome, while also negatively impacts the phospholipid biosynthesis and intestine integrity by decreasing goblet cell count.

*Pseudomonas aeruginosa*, a Gram-negative motile, non-spore-forming bacterium, that is a fish commensal but could be rendered as highly opportunistic and pathogenic under stress and result in serious diseases which include gill necrosis, hemorrhagic septicemia, abdominal distention, friable liver and congested kidney (Ardura et al., 2013). *Pseudomonas* spp. are also associated with food-borne illness in humans through consumption of contaminated food, including raw fish, its byproducts and other types of sea food (De Smet et al., 2017). In certain cases, the colonies of *P. aeruginosa* identified from fish are found to be closely related to the ones which cause hospital-acquired pneumonia in humans (Chevalier et al., 2017). Our previous work, in which zebrafish were exposed to LCs of two different antibiotics from embryonic stage to adulthood, did not suggest presence of *P. aeruginosa* in exposed samples (Kayani et al., 2021). However, in our current study, the results have suggested that exposure to OTC results in increased relative abundance of *P. aeruginosa* in zebrafish gut microbiome. This also suggests differential impact of exposure at early life and at adulthood. Furthermore, we also observed positive association between the duration of exposure and the relative abundance of *P. aeruginosa*. Interestingly, the control groups did not show such trend. Therefore, it is plausible that the exposure to antibiotics in the environments serves as a stress and results in gradual accumulation of *P. aeruginosa* which may render these zebrafish prone to health-associated complications. However, further experiments are required to develop an understanding of the relationships between increased abundance of *P. aeruginosa* and zebrafish health.

In addition to *P. aeruginosa*, several other microbial species were also positively correlated with exposure duration which included *Alicycliphilus denitrificans*, a heterotrophic microbe. *A. denitrificans* has been reported to be highly efficient in degradation of tetracycline and oxytetracycline (Solís-González and Loza-Tavera, 2019). Therefore, it is arguable that the relative abundance of *A. denitrificans* is increased in the zebrafish gut microbiome for accelerating the biodegradation of OTC. Other microbes, significantly enriched after exposure, include *Undibacterium parvum, Fimbriimonas ginsengisoli*, and *Acidovorax carolinesis* are less familiar names in fish gut microbiome, therefore, further experiments are required to reveal their role in the zebrafish gut microbiome after exposure to OTC.

Environmental stress can also serve as selective pressure and mobilize the antibiotic resistance genes from commensals to pathogens or pathogens to commensals (Von Wintersdorff et al., 2016). Our previous study has shown that zebrafish exposed to OTC have significantly high abundance of several tetracycline resistance genes in their gut microbiome (Kayani et al., 2021). Here, we not only characterized the antibiotic resistome of the zebrafish gut but also identified strong correlations between ARGs and MGEs. Relative abundance of multiple tetracycline resistance genes increased after zebrafish were exposed to OTC. These findings are in concordance with different recent reports, which also suggest increased relative abundance of tetracycline resistance genes after exposure to OTC (Ma et al., 2019; Zhang et al., 2019). Longitudinal analysis is cruicial for highlighting the temporal shifts in the abundance of ARGs and their associations with the duration of exposure. Here, the relative abundance of certain tetracycline resistance genes [e.g., *tet(E)*] increased as the exposure time increased. The visualization of this effect was only possible through the longitudinal analysis of zebrafish gut microbiome. The association between ARGs and MGEs was assessed using HAllA which suggested co-occurrence of several tetracycline resistance genes (*tet33, tetA, tetB, tetE, tetP, tetZ*, and *tetX6*) with various MGEs that included *IS91, tniA, tniB*, and *qacEdelta* (Fang et al., 2016). We have previously identified similar associations between ARGs and MGEs, in a different study design (Kayani et al., 2021). Based on these observations, it is plausible that tetracycline resistance genes in the zebrafish gut could become mobile with the help of these MGEs.

The intestinal mucosal barrier, the first line of defense against pathogenic and non-pathogenic microorganisms (Martens et al., 2018), is secreted by the intestinal goblet cells. Pathogenic microorganisms may establish infection by disrupting the mucous layer (Martens et al., 2018). The decrease of intestinal goblet cells may increase the risk of intestinal infection in host as it would allow pathogenic bacteria to enter the blood circulation from the intestine (Knoop and Newberry, 2018; Zhang and Wu, 2020). Phospholipids are highly essential for the normal function of the intestinal mucus barrier (Kennelly et al., 2021). We observed that exposure to OTC resulted in significant reduction of goblet cells in the exposed host whereas the relative abundnace of phospholipid synthesizing genes in the microbiome of the exposed host was also decreased. Kaur et al., have demonstrated that the antibiotics have detrimental effect on the intestinal histology and cause reduction of goblet cell numbers which results in progression of tumor development (Kaur et al., 2018). However, this work was based on the clinical concentrations (1 mg/mL) of multiple different antibiotics (including vancomycin, neomycin and ampicillin) administered to mice models. In contrast, our study involved exposure to a single antibiotic at much lower concentration than the concentration used in clinics. Furthermore, it is well known that the alterations in gut microbiome, immunity and/or mucous membranes often render chronic infectious diseases such as inflammatory bowel disease (IBD), obesity, diabetes, and rheumatoid arthritis (Alemao et al., 2021). This study, to the best of our knowledge, is the first of its kind to analyze the impact of exposure to LCs of an antibiotic on phospholipid biosynthesis pathway and goblet cell counts (of hosts). However, here, we did not evaluate the health-associated implications of altered goblet cell counts and phospholipid biosynthesis. Therefore, to comprehensively understand the connection between microbial phospholipid metabolism, host goblet cell counts and the impact of their disequilibrium on immunity in host, further work is needed.

## Conclusion

In summary, we have proved that exposure to low concentration of OTC in accumulation of pathogenic bacteria and antibiotic resistant genes, and also hampers the ability of intestinal bacteria to synthesize phospholipids and alters goblet cell counts of the zebrafish intestine. Together, these results indicate that even very low concentration of OTC may increase the chance of infections in the exposed and organism and may result in renderring them antibiotic resistant. Therefore, such low concentrations of OTC (or other environmental antibiotics) should be taken seriously and effective methods need to be desgined for avoiding their release in the environment.

## Author's note

MRK is a postdoctoral researcher at the Department of Infectious Diseases, Xinhua Children's Hospital, Xinhua Hospital, Shanghai Jiao Tong University School of Medicine. KY is a graduate student at School of Life Sciences, Fudan University, Shanghai. YQ is a graduate student at the Shanghai Jiao Tong University School of Medicine. XY is a researcher at Ministry of Education and Shanghai Key Laboratory of Children's Environmental Health, Xinhua Hospital, Shanghai Jiao Tong University School of Medicine. LC is a principle investigator at Shanghai Institute of Immunology, Shanghai Jiao Tong University School of Medicine. LH is a principle inversitgator and associate professor at the Department of Infectious Diseases, Xinhua Children's Hospital, Xinhua Hospital, Shanghai Jiao Tong University School of Medicine.

## Data availability statement

The data presented in the study are deposited in the NCBI SRA repository, accession number PRJNA781418.

## Ethics statement

The animal study was reviewed and approved by Ethics Committee of Xinhua Hospital Affiliated to Shanghai Jiao Tong University School of Medicine.

## Author contributions

MK, KY, and YQ contributed to the conceptualization, data curation, formal analysis, methodology, and editing of the manuscript. XY assisted in the project administration. LC supervised library preparation, sequencing, and data analysis. LH contributed to the conceptualization, funding acquisition, supervision, and review for the entire project. All authors read and approved the final manuscript.

## Funding

This work was supported by the National Natural Science Foundation of China (No. 81874265), National Natural Science Foundation of China (No. 82073561), Shanghai Science and Technology Commission (No. 18411966600), Shanghai Science and Technology Commission (No. 19410740800), and Shanghai Jiao Tong University School of Medicine (No. 2020002).

## Conflict of interest

The authors declare that the research was conducted in the absence of any commercial or financial relationships that could be construed as a potential conflict of interest.

## Publisher's note

All claims expressed in this article are solely those of the authors and do not necessarily represent those of their affiliated organizations, or those of the publisher, the editors and the reviewers. Any product that may be evaluated in this article, or claim that may be made by its manufacturer, is not guaranteed or endorsed by the publisher.

## Abbreviations

OTC: Oxytetracycline
LCs: Low concentrations
ARGs: Antibiotic resistance genes
MGEs: Mobile genetic elements
HGT: Horizontal gene transfer
Dpf: Days post-fertilization
HAllA: Hierarchical All-against-All association
LDA: Linear discriminant analysis
FDR: False discovery rate.
