# Supplementary material for: Longitudinal analysis of exposure to a low concentration of oxytetracycline on the zebrafish gut microbiome
Source: Front Microbiol. 2022 Sep 22;13:985065. doi: 10.3389/fmicb.2022.985065 (PMC9536460; doi:10.3389/fmicb.2022.985065)
Supplement: Supplementary file 1 [file Data_Sheet_1.DOCX]

# Supplementary Information

# Longitudinal analysis of exposure to a low concentration of oxytetracycline on the zebrafish gut microbiome

Masood ur Rehman Kayani^1,2*^, Kan Yu^1,3*^, Yushu Qiu^1*^, Xiaogang Yu^4^, Lei Chen ^5^, Lisu Huang^1,2+^

1 Department of Infectious Diseases, Xinhua Children’s Hospital, Xinhua Hospital, Shanghai Jiao Tong University School of Medicine, Shanghai 200092, China

2 The Children's Hospital, Zhejiang University School of Medicine, National Clinical Research Center for Child Health, Hangzhou 310052, China

3 School of Life Sciences, Fudan University, Shanghai 200438, China

4 Ministry of Education and Shanghai Key Laboratory of Children’s Environmental Health, Xinhua Hospital, Shanghai Jiao Tong University School of Medicine, Shanghai 200092, China

5 Shanghai Institute of Immunology, Shanghai Jiao Tong University School of Medicine, Shanghai 200025, China

* These authors contributed equally

+ To whom correspondence should be addressed: Lisu Huang (huanglisu@xinhuamed.com.cn)

**
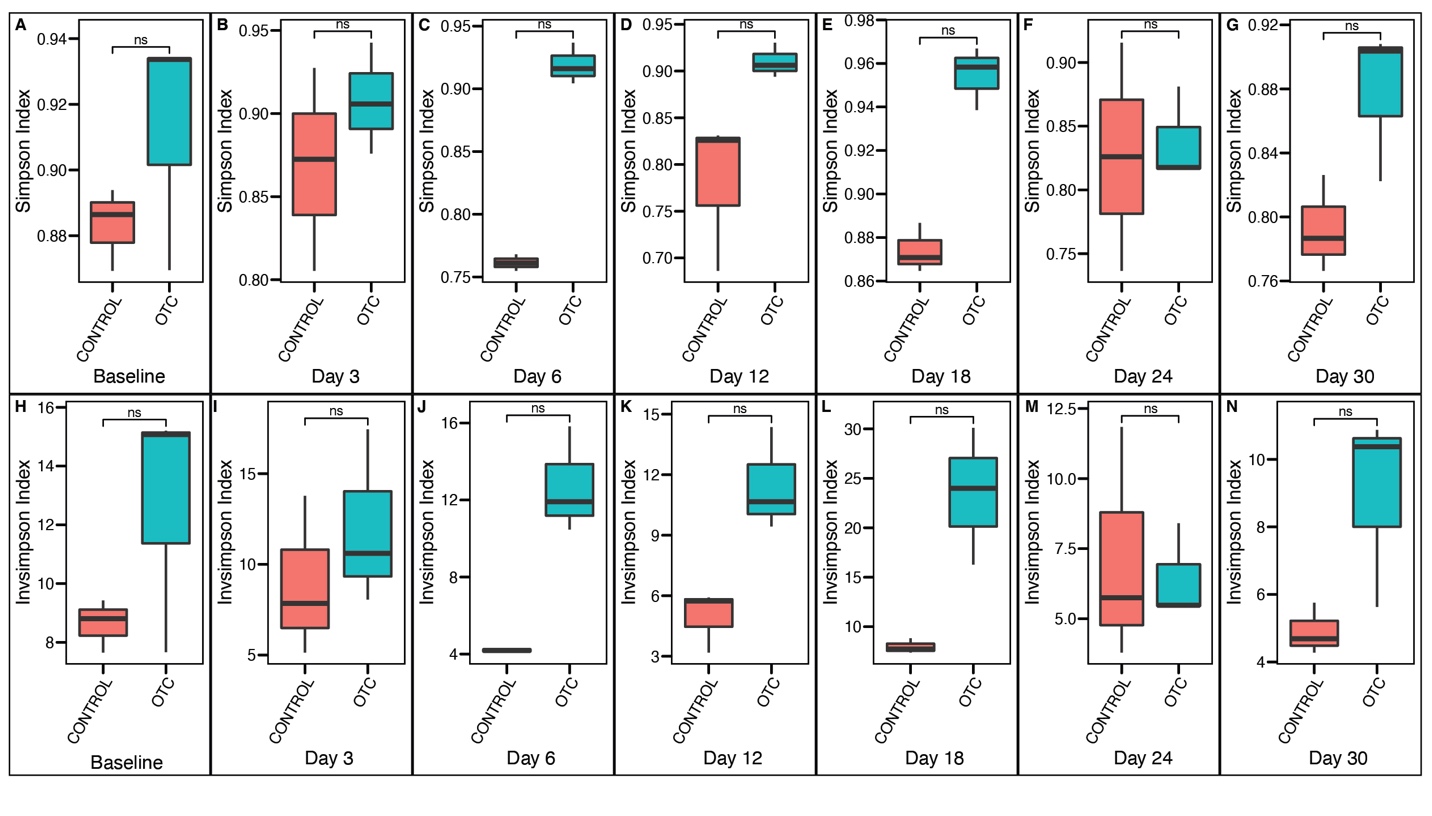
**

**Supplementary Figure 1: Comparisons of alpha diversity indices (Simpson and inverse Simpson index) between Control and OTC exposure groups at different timepoints.**

**
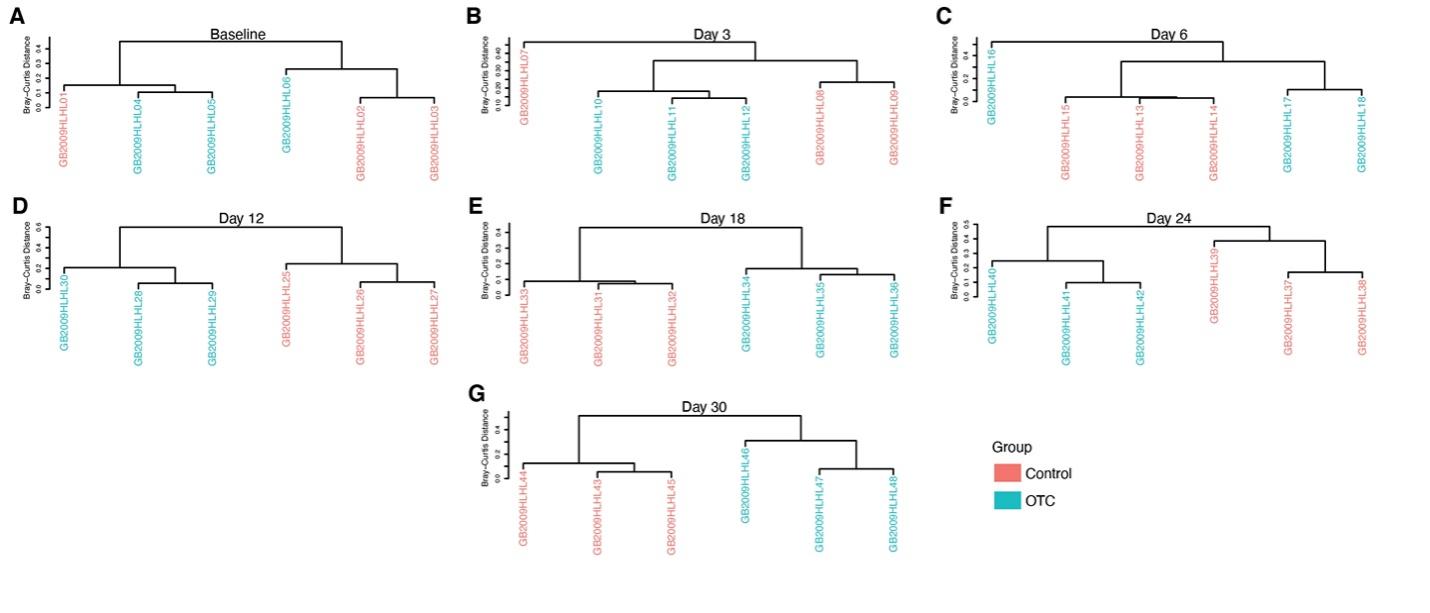
**

**Supplementary Figure 2: Cluster analysis of control and OTC exposed samples based on Bray-Curtis dissimilarities computed for Species compositions**

**
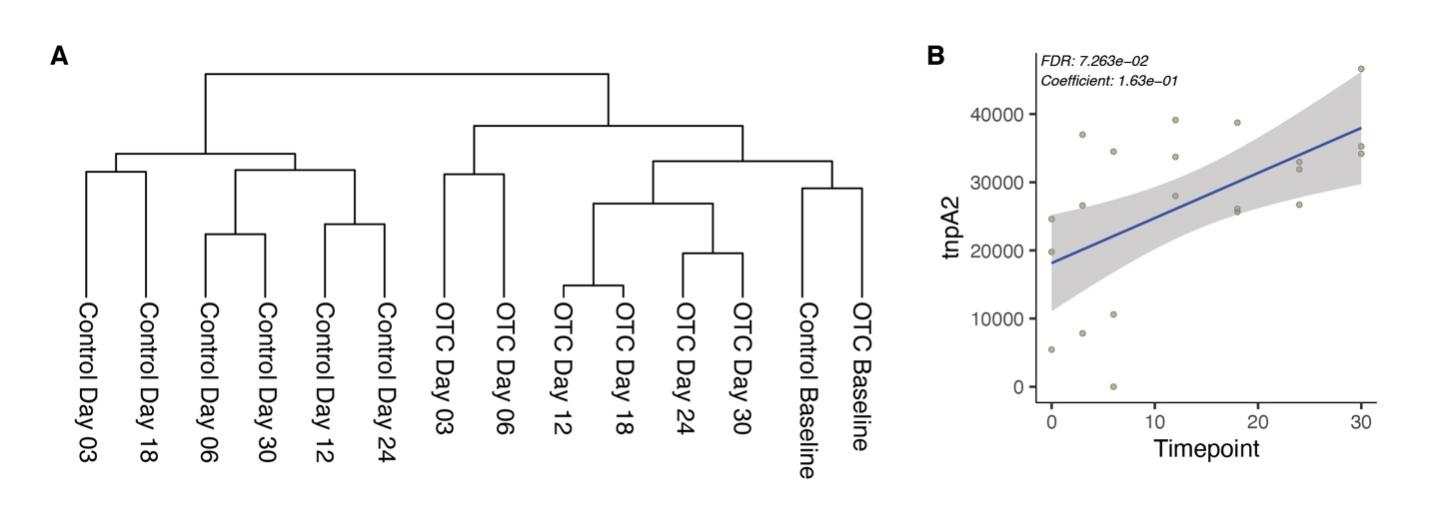
**

**Supplementary Figure 3: Dendrogram computed for the composition of MGEs (A) and positive correlation of tnpA2 gene in the OTC exposure group (B).**
